# Thoracic angle of trunk rotation and its association with myopia among schoolchildren in a rural county of northeastern China

**DOI:** 10.3389/fpubh.2026.1836894

**Published:** 2026-08-06

**Authors:** Tianli Zheng, Yunlong Bi, Shuang Jiang, Zhijie Ren, Yang Zhang, Weiwei Fu

**Affiliations:** 1 Medical Imaging Department, Suzhou Institute of Biomedical Engineering and Technology, Chinese Academy of Sciences, Suzhou, Jiangsu, China; 2 Department of Orthopaedic Surgery, The First Affiliated Hospital of Jinzhou Medical University, Jinzhou, Liaoning, China; 3 Collaborative Innovation Center for Health Promotion for Children and Adolescents, Jinzhou, Liaoning, China; 4 Ophthalmology Center, The Third Affiliated Hospital of Jinzhou Medical University, Jinzhou, Liaoning, China

**Keywords:** cross-sectional study, myopia, postural asymmetry, school-aged children, thoracic angle of trunk rotation

## Abstract

**Objective:**

Myopia and poor spinal health are prevalent among schoolchildren. We hypothesized that subclinical postural asymmetry, measured by the thoracic angle of trunk rotation (ATR), is positively associated with myopia in schoolchildren from a rural county in northeastern China.

**Methods:**

This cross-sectional study evaluated 2,599 schoolchildren (grades 1–6) recruited from two primary schools in a single rural county of northeastern China. We measured thoracic ATR using a scoliometer and assessed refractive error. The association between ATR and myopia was analyzed using multivariable logistic regression, restricted cubic spline-based exposure–response modeling, and machine learning for feature importance.

**Results:**

Among participants (51.0% boys, 49.0% girls; mean age 9.5 ± 1.7 years), the overall myopia prevalence was 52.8%. After adjusting for confounders, 1° increase in thoracic ATR was associated with 7% higher odds of myopia (odds ratio = 1.07). Children in the highest ATR tertile had significantly increased odds of myopia compared to the lowest. A linear association was identified. Furthermore, feature importance analysis ranked thoracic ATR as a leading marker for myopic status, secondary only to age and gender.

**Conclusion:**

Elevated subclinical thoracic ATR is independently associated with myopia. As a concurrent epidemiological marker of poor posture, ATR screening supports integrated *vision-spine* health initiatives for children in resource-limited settings.

## 1 Introduction

Myopia has emerged as a pressing global public health challenge, with particularly high burdens reported among school-aged populations in high-prevalence Asian regions, including China, Japan, Singapore, and South Korea (1–4). In rural China, where screening resources may be more limited, posture-related risks may be amplified. Projections indicate that nearly half of the world’s population will be affected by myopia by 2050 (1), imposing substantial social and economic burdens on families and healthcare systems. Beyond impairing visual acuity, high myopia is a major risk factor for blinding ocular conditions such as retinal detachment and glaucoma (5, 6).

The International Myopia Institute (IMI) identifies prolonged near-work and insufficient outdoor activity as key environmental drivers of myopia onset and progression (7, 8), behaviors often accompanied by sedentary sitting that also threaten children’s musculoskeletal health (9). Spinal health issues among school-aged children are equally concerning, and poor reading and writing ergonomics are increasingly recognized as important risk factors for musculoskeletal complaints and early postural deviations (10).

Emerging research has explored links between myopia and clinical spinal structural curvatures, such as adolescent idiopathic scoliosis. Some studies reported a higher risk of scoliosis in myopic students (11), and a recent systematic review further confirmed that myopic children have a significantly higher incidence of scoliosis than their normal-sighted peers (12). However, these findings are not universal, highlighting an unresolved ambiguity in the relationship between myopia and musculoskeletal health (13). Notably, prior research has predominantly focused on diagnosed, severe scoliosis (typically defined by a Cobb angle ≥10°), often neglecting early, subclinical postural asymmetry. Furthermore, the causal sequence remains heavily debated; rather than a simple one-way etiology, recent evidence suggests a complex, potentially bidirectional relationship. Visual impairments like early-onset myopia may induce compensatory postural shifts (14), while poor ergonomics simultaneously stress both the visual and musculoskeletal systems.

Recent Asian school-based evidence has further extended this line of inquiry. In a large junior-middle-school cohort from Shanghai (*n* = 9,583), scoliosis screening positivity and poor reading/writing posture were each independently associated with comorbid myopia and scoliosis (15). A pediatric health-examination study that jointly recorded spinal alignment parameters and refractive status additionally reported that a more myopic spherical equivalent was correlated with a higher angle of trunk rotation, suggesting that the link between refractive error and trunk asymmetry may be detectable even in the subclinical range (16). These observations indicate that the myopia–spine connection in school-aged children is unlikely to be confined to clinically diagnosed scoliosis, and that early postural indicators such as ATR may carry independent epidemiological information.

The thoracic angle of trunk rotation (ATR) is a quantitative, non-invasive measure obtained using a Bunnell scoliometer, sensitively reflecting trunk rotational asymmetry (17). In healthy children, thoracic ATR is typically low, but minor elevations can serve as the earliest morphological footprints of poor postural habits before structural deformity occurs (18). Thoracic ATR values in the present study sample were predominantly within the subclinical range and below commonly used scoliometer referral thresholds for further orthopaedic evaluation, such as 5°–7° (17, 19). The present study therefore focused on subclinical thoracic rotational asymmetry rather than clinically suspected scoliosis. To date, no studies have investigated the quantitative association between myopia and subclinical thoracic ATR, despite theoretical plausibility from shared risk factors.

Investigating this relationship carries significant public health implications. Both conditions are highly prevalent among school-aged children, and their potential link at the subclinical stage could inform integrated *vision-spine* screening strategies, particularly valuable in rural China, where dual health burdens may be exacerbated by limited screening resources. Therefore, this study used school-based data from a rural county in northeastern China to explore the concurrent association between myopia and thoracic ATR, characterize its exposure–response pattern, and offer new evidence for integrated child health management.

## 2 Methods

### 2.1 Study design and subjects

This school-based cross-sectional epidemiological study was conducted in Yixian County, Liaoning Province, China, in April 2023. The study subjects were all students from grades 1 to 6 in two local primary schools. After obtaining written informed consent from the guardians of all participating students, a total of 3,445 students were recruited. This study protocol was approved by the Ethics Committee of the Third Affiliated Hospital of Jinzhou Medical University (Ethical Approval No.: JYDSY-KXYJ-IEC-2023-018). The study was conducted in accordance with the Declaration of Helsinki and reported following the STROBE statement (20).

Inclusion criteria: Students in grades 1–6 in school, and written informed consent obtained from their guardians.

Exclusion criteria: (1) Those who failed to participate in the screening for various reasons (*n* = 32); (2) Those who could not cooperate to complete the ophthalmologic or physical examination; (3) Those with missing key data (refractive error, thoracic ATR) (*n* = 809); (4) Those with internally inconsistent or biologically implausible records, such as impossible anthropometric values or apparent data-entry inconsistencies (*n* = 5). Finally, a total of 2,599 students with complete data were included in the final analysis.

To characterize the nature of missing data, we compared baseline characteristics between participants with complete data (*n* = 2,599) and those excluded due to missing key variables (*n* = 809). No significant difference was observed in sex distribution between the two groups (*p* = 0.759). However, excluded participants were significantly younger (mean age 8.61 vs. 9.73 years, *p* < 0.001), with lower-grade students (Grades 1–2) being disproportionately represented among those with missing thoracic ATR measurements. This pattern is consistent with the well-recognized difficulty of obtaining reliable scoliometer readings in younger, less cooperative children, rather than reflecting a systematic bias related to visual status. The sex-balanced exclusion pattern further supports that the missing data is unlikely to have introduced directional bias into the association estimates reported here.

### 2.2 Key terminology definitions and measurements

Myopia: Defined based on autorefraction results without cycloplegia. Non-cycloplegic refraction may overestimate myopia prevalence because of residual accommodation, particularly in younger children, and the absence of cycloplegic confirmation should be regarded as a recognized limitation that may modestly affect the generalizability of the prevalence estimates reported here. To reduce potential misclassification, a more stringent threshold of spherical equivalent (SE) ≤ −0.75 D was adopted rather than the conventional −0.50 D cutoff (21), in line with the IMI recommendation for non-cycloplegic surveys in younger populations (22) and the receiver operating characteristic (ROC)-based optimal cutoff reported by Senoo et al. (23) (sensitivity 90.5%, specificity 95.7%). This threshold has also been adopted in recent large-scale Chinese school-based studies, including the Weifang study involving more than one million students (24). All statistical analyses were based on right eye data to avoid the impact of inter-eye data correlation on the results. The formula for SE is: SE = spherical power + 1/2 cylindrical power.

Thoracic ATR: Measured using the Bunnell scoliometer (17). The subject bent forward at the waist at 90°, with arms hanging naturally. The examiner placed the scoliometer horizontally on the subject’s back and slid it along the spine from top to bottom, recording the maximum reading in the thoracic T2–T5 segment, in degrees (°). The T2–T5 thoracic segment was selected as the primary exposure region because this study focused on upper thoracic rotational asymmetry measured during the forward-bending scoliometer examination. This region may be relevant to cervicothoracic postural changes during reading and writing. Other spinal segments were not included as primary exposures to avoid multiple segment-wise comparisons in this exploratory epidemiological analysis. This index quantifies the degree of rotational asymmetry of the trunk in the horizontal plane and is an important indicator for early scoliosis screening. The Bunnell scoliometer has well-documented measurement properties for trunk rotation assessment (17, 18). Prior to this study, an internal methodological pilot conducted by our team in 41 adult volunteers, using the identical standardized protocol, yielded an intra-examiner intraclass correlation coefficient (ICC) of 0.82 (95% CI: 0.72–0.89), supporting procedural repeatability. All field examiners participating in the present screening were trained under this same standardized protocol to ensure consistency of measurement. At the screening stage, signed thoracic ATR values were recorded to encode the direction of rotation (i.e., the laterality of the rib prominence on forward bending, corresponding to clockwise vs. counterclockwise rotation); the absolute magnitude (|ATR|) was used as the primary analytic variable, while the signed values were retained for a sensitivity analysis.

Body Mass Index (BMI): Calculated using the formula “weight (kg)/[height (m)]^2^.”

Ophthalmologic examinations were performed using an autorefractor compliant with ISO 10342 standards (ARK7800L/M, Shanghai Chang E, China). Anthropometric data (height, weight, waist circumference, hip circumference) were measured by trained professionals using standardized equipment, with the average value taken after three repetitions for each measurement.

### 2.3 Statistical methods

Participants were classified based on their myopia status. Descriptive statistics for continuous variables are presented as mean ± standard deviation (Mean ± SD) for normally distributed data, and as median (interquartile range) for non-normally distributed data. Group comparisons for categorical variables were performed using chi-square tests, and comparisons for continuous variables were made using the independent t-test for normally distributed data and the Mann–Whitney *U* test for non-normally distributed data. The normality of continuous variables was assessed using the Shapiro–Wilk test.

To assess the relationship between Thoracic ATR (analyzed as both continuous and categorical variables) and myopia, multivariable logistic regression models were used for myopia analysis (binary outcome). These models adjusted for potential confounders using three different stratification methods: Model 1 (Adjusted only for gender), Model 2 (Further adjusted for age and ethnicity), and Model 3 (fully adjusted model including gender, age, ethnicity and BMI). Waist circumference and hip circumference were not included in Model 3 alongside BMI because preliminary variance inflation factor (VIF) checks indicated substantial multicollinearity among these anthropometric indicators (waist VIF = 5.12; hip VIF = 6.00). After retaining BMI as the sole anthropometric covariate, all VIFs in the Model 3 were below 2, indicating no concerning multicollinearity (Supplementary Table 1).

In all primary models, thoracic ATR was entered as its absolute magnitude (|ATR|), because the prespecified scientific question concerned the degree of subclinical trunk rotational asymmetry rather than its laterality. To verify that this analytic choice did not obscure a directional effect, a sensitivity analysis was performed using the original signed thoracic ATR records, in which (i) direction alone (positive vs. negative rotation), (ii) the |ATR| × direction interaction in the fully adjusted model, and (iii) the |ATR| effect within each rotation-direction subgroup were estimated, adjusting for gender, age, ethnicity, and BMI.

To evaluate whether pubertal maturation might confound the observed thoracic ATR–myopia association, an additional sensitivity analysis was conducted using sex-specific chronological-age proxies for pubertal maturation. Because Tanner stage, menarcheal status, spermarche, and peak height velocity were not collected, these proxies were not interpreted as definitive classifications of individual pubertal status, but as progressively conservative approximations of a pre-pubertal sample. Guided by published Chinese and international data on the timing of pubertal onset (breast development and menarche in girls; testicular enlargement and spermarche in boys) (25–29), we constructed four subsets by applying increasingly restrictive age ceilings, thereby excluding children who were progressively more likely to have entered puberty: a lenient subset (girls <12 y/boys <13 y), which excluded mainly children likely to be in late puberty; a moderate subset (girls <11 y/boys <12 y); a stricter subset (girls <10 y/boys <11 y), approximating the age below which most children in these populations have not yet reached pubertal onset; and an exploratory very-young subset (girls <8 y/boys <9 y), representing a highly conservative, almost certainly pre-pubertal group. These four labels denote the degree of age restriction and correspond to the subsets defined in Supplementary Table 4. Model 3 was re-estimated within each subset and compared with the full-sample result.

A restricted cubic spline (RCS) curve with 4 knots was used to investigate the exposure–response relationship between Thoracic ATR and the risk of myopia. Interaction analyses were conducted to examine the effects of Thoracic ATR on myopia across different subgroups defined by gender, ethnicity, age, and BMI. The results were visually presented using forest plots, which illustrated the relative effects and confidence intervals for each subgroup. *p*-values for the interaction effects were obtained by likelihood ratio test.

We also evaluated model performance and interpreted the specific contribution of Thoracic ATR to myopic status. The goodness-of-fit of this model was first evaluated using ROC curves, assessing the model’s discriminative ability. Next, the calibration curve was used to assess the consistency between estimated probabilities and observed outcomes. Subsequently, the SHapley Additive exPlanations (SHAP) model was applied to interpret the importance of Thoracic ATR and other variables in identifying myopia, based on the constructed model.

All statistical analyses were performed using FreeStatistics (version 2.4.0) and R software (version 4.2.2). The relationship between the study variables and myopia was reported using odds ratios (OR) with 95% confidence intervals (CI). A *p*-value of less than 0.05 was considered statistically significant.

## 3 Results

### 3.1 Population characteristics

**Figure 1:**
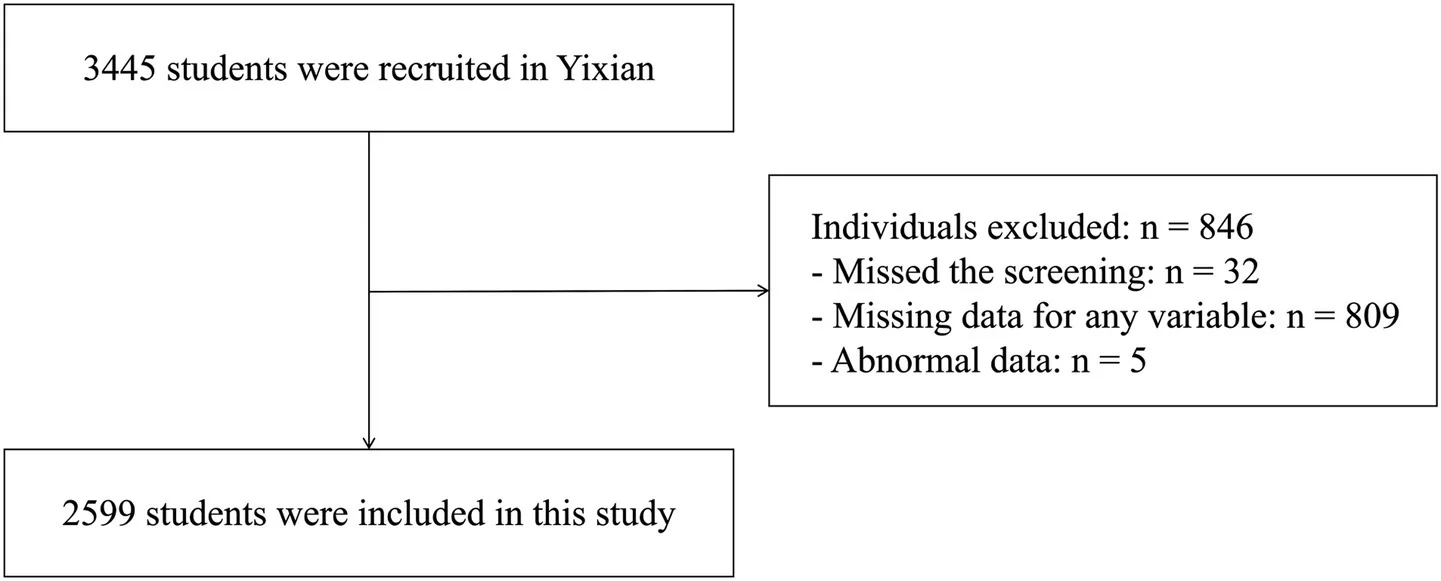
Study population flowchart. The diagram illustrates the participant recruitment and exclusion process in Yixian. Starting from 3,445 students initially recruited, 846 were excluded due to missed screenings, missing variable data, or abnormal data, resulting in a final sample of 2,599 participants included in the analysis.

The study included a total of 2,599 participants (Figure 1), stratified into two groups based on myopia status: those without myopia (*N* = 1,226) and those with myopia (*N* = 1,373). The mean age was 9.5 ± 1.7 years, and 1,274 participants (49.0%) were female. Thoracic ATR values were predominantly subclinical: 97.4% of children had thoracic ATR values below 5°, and 99.3% had values below 7°. The median thoracic ATR was 1.34° overall and was higher in children with myopia than in those without myopia (1.41° vs. 1.24°; *p* < 0.001). As shown in Table 1, significant differences were observed between the two groups in baseline characteristics. Specifically, older age, Han ethnicity, larger waist and hip circumferences, higher BMI, and greater Thoracic ATR were observed in the myopia group compared to the non-myopia group. Furthermore, results indicated that female gender was associated with a higher likelihood of myopia. The detailed results of the univariate analysis for all baseline characteristics are provided in Supplementary Table 2.

**Table 1:** Characteristics of participants by myopia category.

Characteristic	All participants	With myopia		*P-*value	Effect size
		No	Yes		
*N*	2,599	1,226	1,373		
Gender, *n* (%)				<0.001	OR = 1.40 (1.20–1.63)
Male	1,325 (51.0)	679 (55.4)	646 (47.1)		
Female	1,274 (49.0)	547 (44.6)	727 (52.9)		
Age, year	9.5 ± 1.7	8.9 ± 1.7	9.9 ± 1.5	< 0.001	Cohen’s *d* = 0.62
Spherical equivalent, *D*	−0.8 (−1.9, −0.1)	0.0 (−0.4, 0.2)	−1.8 (−2.8, −1.1)	< 0.001	r = 0.82
Ethnicity, *n* (%)				0.029	Cramér’s *V* = 0.05
Han	861 (33.1)	380 (31)	481 (35)		
Manchu	1,357 (52.2)	660 (53.8)	697 (50.8)		
Others^a^	381 (14.7)	186 (15.2)	195 (14.2)		
Waist circumference, cm	69.0 ± 10.5	67.9 ± 10.3	69.9 ± 10.5	<0.001	Cohen’s *d* = 0.19
Hip circumference, cm	80.8 ± 11.2	78.9 ± 11.2	82.5 ± 11.1	<0.001	Cohen’s *d* = 0.32
BMI, kg/m^2^	20.6 ± 4.7	20.4 ± 4.7	20.8 ± 4.7	0.016	Cohen’s *d* = 0.09
Thoracic ATR, °	1.3 (0.7, 2.2)	1.2 (0.6, 2.1)	1.4 (0.7, 2.3)	<0.001	*r* = 0.07
Thoracic ATR group				0.003	Cramér’s *V* = 0.06
Less than 0.86	865 (33.3)	448 (36.5)	417 (30.4)		
0.86 to 1.86	864 (33.2)	396 (32.3)	468 (34.1)		
More than 1.86	870 (33.5)	382 (31.2)	488 (35.5)		

Categorical variables are reported as *N* (%) and continuous variables are reported as mean (SD) or median (T1, T3). Statistical tests: *χ*^2^ = chi-square test; *t*-test = independent-samples *t*-test; Mann–Whitney *U* = Wilcoxon rank-sum test. Effect-size estimates: OR (95% CI) for categorical 2 × 2 comparisons, Cohen’s *d* for normally distributed continuous variables, *r* for non-normally distributed continuous variables, and Cramér’s *V* for categorical variables with >2 categories.

BMI, body mass index; ATR, angle of trunk rotation. ^a^includes Xibe, Mongolian, Chinese Korean, Tujia, Daur, Yi, Tibetan, Zhuang nationality.

### 3.2 Association between thoracic ATR and myopia

Thoracic ATR was analyzed both as a continuous variable and as sample-specific tertile categories in Table 2. For the categorical analysis, thoracic ATR was divided into tertiles based on its distribution: T1 (<0.86), T2 (0.86–1.86), and T3 (>1.86). These ATR tertile cut-points were sample-specific exposure categories and should not be interpreted as universal clinical thresholds or myopia-risk cut-offs. We applied logistic regression models to investigate the associations between Thoracic ATR and myopia (Table 2).

**Table 2:** Odds ratio of association between thoracic ATR and myopia in multivariable logistic regression analysis.

Variable	*N*	Event (%)	Crude		Model 1		Model 2		Model 3	
			OR (95% CI)	*P-*value	OR (95% CI)	*P-*value	OR (95% CI)	*P-*value	OR (95% CI)	*P-*value
Thoracic ATR	2,599	1,373 (52.8)	1.11 (1.05 ~ 1.18)	<0.001	1.11 (1.05 ~ 1.18)	<0.001	1.07 (1.01 ~ 1.14)	0.025	1.07 (1.01 ~ 1.14)	0.023
Thoracic ATR group										
Less than 0.86	865	417 (48.2)	Reference		Reference		Reference		Reference	
0.86 to 1.86	864	468 (54.2)	1.27 (1.05 ~ 1.53)	0.013	1.28 (1.06 ~ 1.54)	0.012	1.19 (0.98 ~ 1.46)	0.080	1.19 (0.98 ~ 1.45)	0.089
More than 1.86	870	488 (56.1)	1.37 (1.14 ~ 1.66)	0.001	1.38 (1.14 ~ 1.67)	0.001	1.22 (1 ~ 1.49)	0.052	1.23 (1.01 ~ 1.5)	0.047

OR (95% CI) values represent effect-size estimates from logistic regression models; *P*-values are derived from Wald tests.

ATR, angle of trunk rotation, measured by the Bunnell scoliometer; OR, odds ratio; CI, Confidence interval; BMI, body mass index.

Model 1 adjusted for gender; model 2 adjusted for gender, age, ethnicity; model 3 adjusted for gender, age, ethnicity, and BMI.

**Figure 2:**
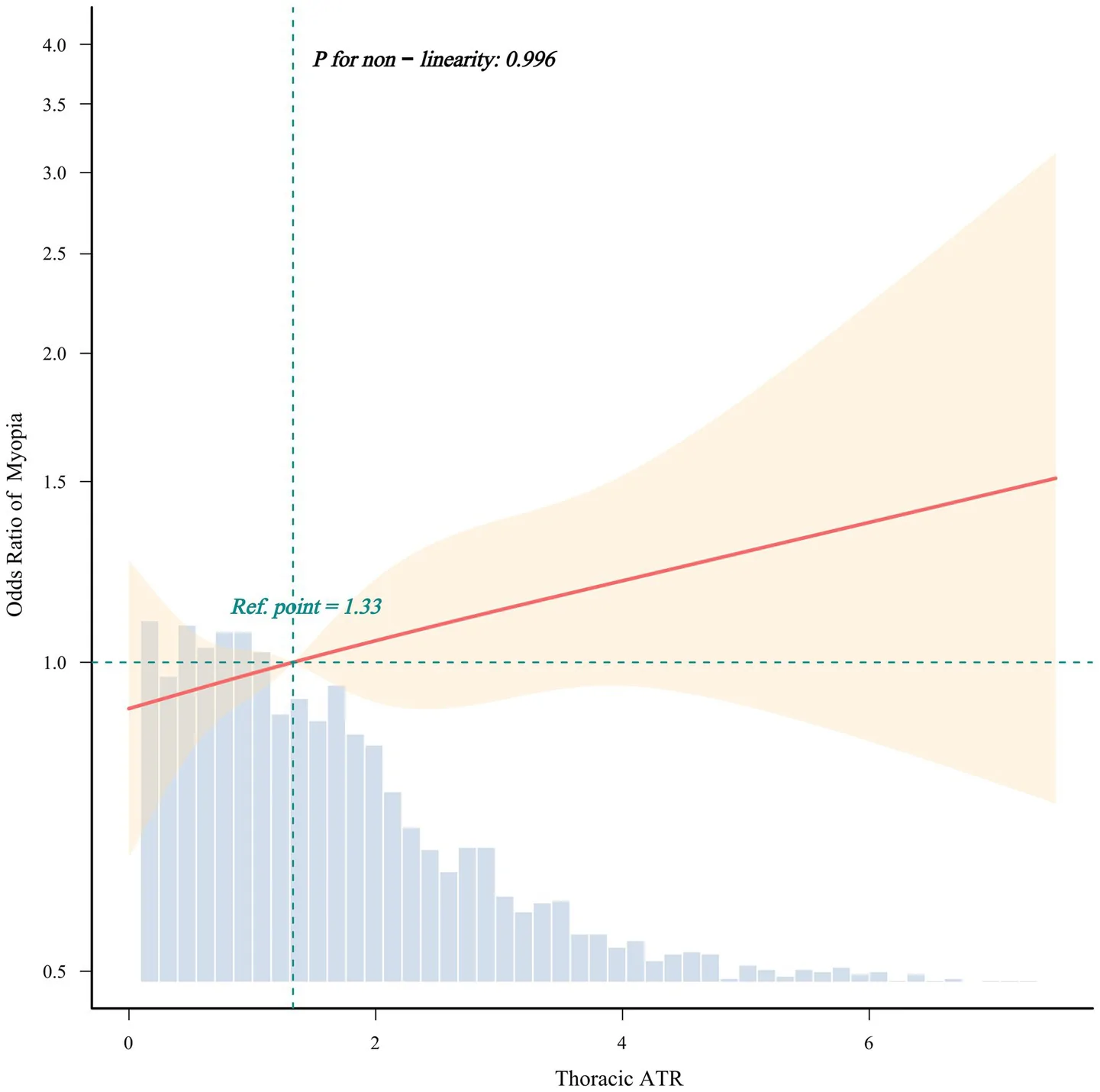
Restricted cubic spline analysis of the association between thoracic angle of trunk rotation (ATR) and the odds ratio of myopia. The restricted cubic spline (RCS) model visualizes the association between thoracic ATR and the probability of myopia. The solid red line represents the adjusted odds ratio, and the shaded area represents the 95% confidence intervals. The model adjusted for gender, age, ethnicity, and body mass index (BMI). The analysis indicates a linear positive association (*P* for non-linearity = 0.996) with a reference point at 1.33.

In the fully adjusted model (Model 3), Thoracic ATR was significantly positively associated with higher odds of myopia. Specifically, each degree increase in Thoracic ATR was associated with an approximately 7% increase in the odds of myopia (OR = 1.07, 95% CI: 1.01–1.14, *p* = 0.023). Further analysis using categorical variables (tertiles) revealed consistent trends. Compared to T1, participants in T3 showed significantly higher odds of myopia (OR = 1.23, 95% CI: 1.01–1.5, *p* = 0.047). After adjusting for potential confounders, the RCS analysis was performed to assess the relationship between Thoracic ATR and the probability of myopia. As shown in Figure 2, a linear positive association was observed, with no evidence supporting a non-linear relationship (nonlinear *p* = 0.996). These results suggest that the odds of myopia increase linearly with the elevation of Thoracic ATR.

### 3.3 Subgroup and interaction analyses

**Figure 3:**
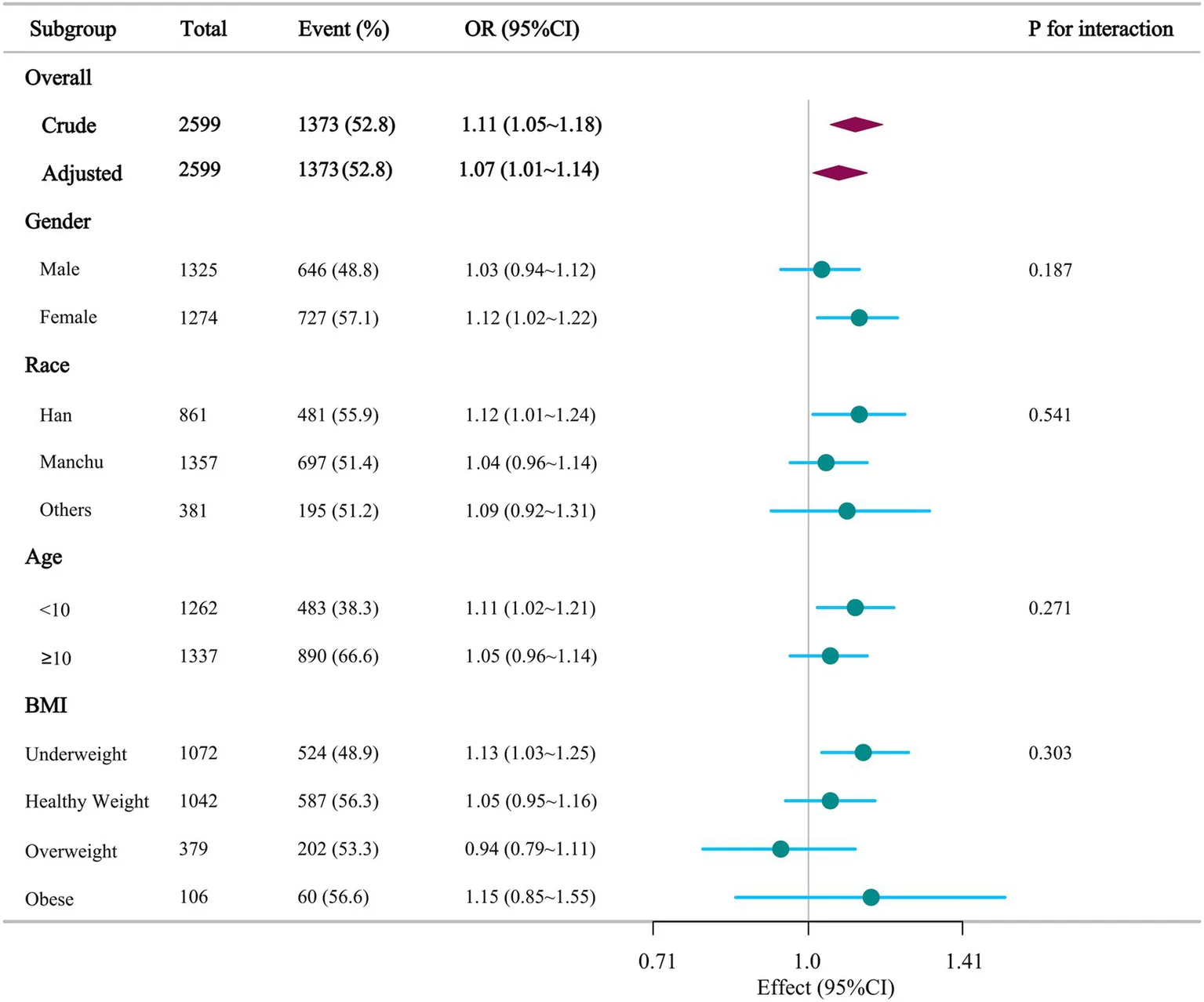
Subgroup analysis of the association between thoracic angle of trunk rotation (ATR) and myopia. Forest plot showing the odds ratios (*OR*) and 95% confidence intervals (*CI*) for the association between thoracic ATR and myopia across different subgroups [gender, ethnicity, age, body mass index (BMI)]. The analyses were adjusted for covariates (age, gender, ethnicity, and BMI), excluding the stratifying variable itself. No significant interactions were observed (all *P* for interaction > 0.05).

To assess the stability of the association between Thoracic ATR and myopia, we performed stratified analyses by gender, ethnicity, age, and BMI (Figure 3). The positive association between Thoracic ATR and myopia appeared statistically significant in specific subgroups, particularly among females (OR = 1.12, 95% CI: 1.02–1.22) and Han ethnicity students (OR = 1.12, 95% CI: 1.01–1.24). However, interaction tests indicated that these differences were not statistically significant (*P* for interaction > 0.05 for all subgroups). Although the ATR–myopia association appeared more pronounced among female students, the interaction by sex was not statistically significant. Therefore, this subgroup pattern should be interpreted cautiously and does not by itself establish a true sex-specific ATR effect. Similar caution applies to the other subgroup findings because all interaction tests were non-significant.

A sensitivity analysis using the original signed thoracic ATR records (*n* = 2,596 after excluding three children with exactly zero rotation) showed that rotation direction alone was not associated with myopia (OR = 0.96, 95% CI: 0.82–1.14, *p* = 0.661), the |ATR| × direction interaction was not significant (interaction OR = 0.98, 95% CI: 0.87–1.11, *p* = 0.805), and the per-degree |ATR| effect was directionally consistent across positive- and negative-rotation subgroups (OR = 1.06 vs. 1.08; Supplementary Table 3). The ATR–myopia association therefore reflects the magnitude of rotational asymmetry rather than its laterality, supporting the use of |ATR| in the primary model.

An additional sensitivity analysis using sex-specific chronological-age proxies for pubertal maturation is presented in Supplementary Table 4. Compared with the full-sample estimate (OR = 1.07, 95% CI: 1.01–1.14, *p* = 0.023), the per-degree OR for thoracic ATR remained positive across all four subsets (range 1.04–1.09). The association retained statistical significance in the lenient (OR = 1.09, 95% CI: 1.02–1.16, *p* = 0.012) and moderate (OR = 1.09, 95% CI: 1.02–1.17, *p* = 0.018) subsets. In the two most restrictive subsets, however, the association was no longer statistically significant, with slightly lower point estimates and higher *p*-values (stricter subset OR = 1.07, 95% CI: 0.99–1.17, *p* = 0.083; exploratory very-young subset OR = 1.04, 95% CI: 0.91–1.19, *p* = 0.565). This attenuation coincided with a marked reduction in sample size and myopia events, from 2,599 participants and 1,373 events in the full sample to 618 participants and 183 events in the very-young subset, and the confidence intervals widened accordingly. Both restricted confidence intervals remained compatible with the full-sample estimate. Because the point estimates also decreased in these two subsets, and because the subsets were nested rather than independent, these analyses cannot distinguish reduced precision from possible age-related attenuation. In the primary subgroup analysis, however, the interaction between age subgroup and thoracic ATR was non-significant (*P* for interaction = 0.271; Figure 3), and the estimate among participants aged <10 years (OR = 1.11, 95% CI: 1.02–1.21) was not lower than that among those aged ≥10 years (OR = 1.05, 95% CI: 0.96–1.14), providing no formal statistical evidence that the association differed by age. Overall, these analyses do not indicate that the full-sample association was solely attributable to pubertal maturation, although possible age-related effect modification cannot be excluded.

### 3.4 Model performance and calibration

**Figure 4:**
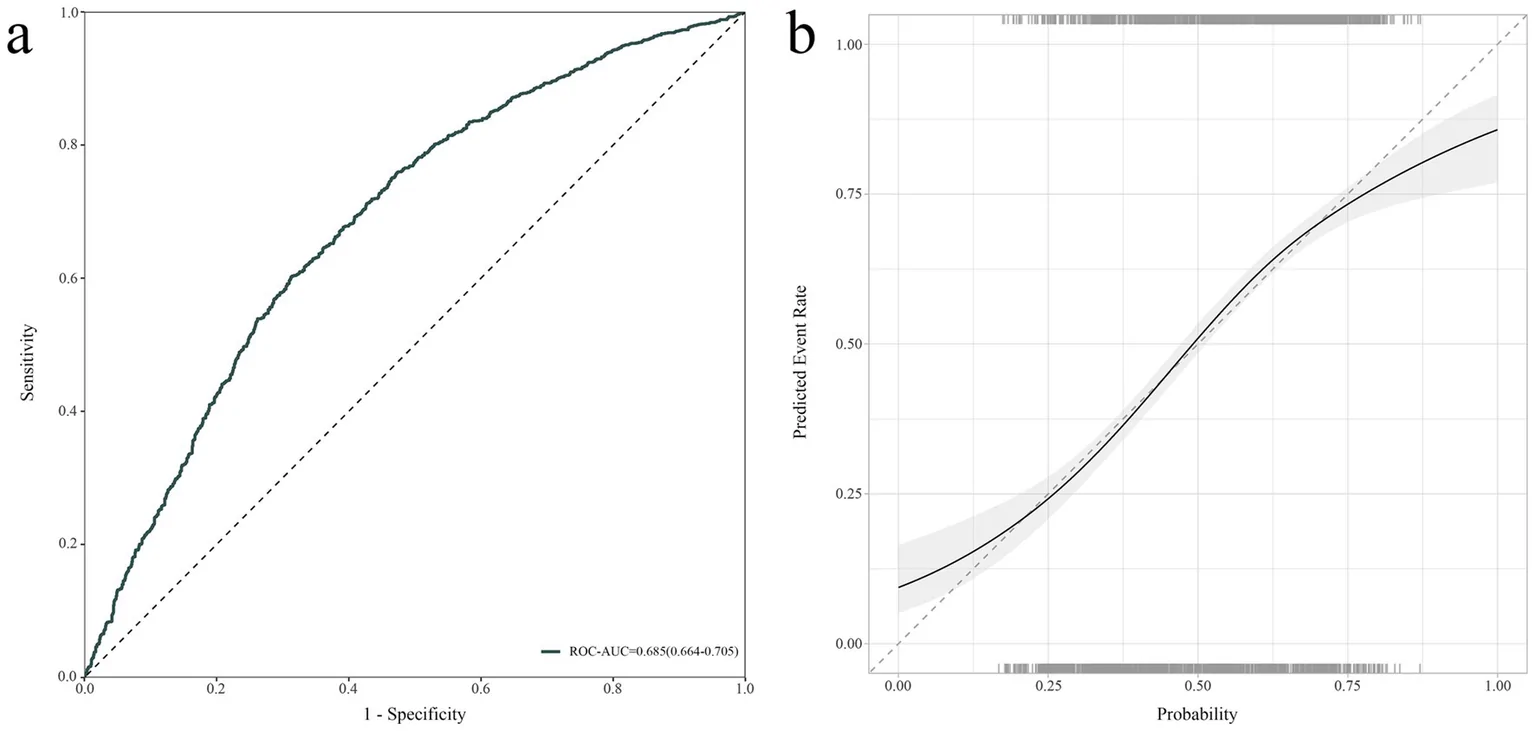
Assessment of model goodness-of-fit **(a)** Receiver Operating Characteristic (ROC) curve illustrating the discriminative ability of the logistic regression model constructed using the entire dataset. The Area Under the Curve (AUC) is 0.685 (95% confidence intervals (CI): 0.664–0.705), indicating modest discriminative ability. **(b)** Calibration curve assessing the agreement between estimated probabilities and observed frequencies of myopia. The curve closely follows the ideal diagonal line (gray dashed line), suggesting good calibration accuracy.

To further validate the discriminative value of Thoracic ATR, a logistic regression model was evaluated. The ROC curve analysis (Figure 4a) yielded an Area Under the Curve (AUC) of 0.685 (95% CI: 0.664–0.705), indicating modest discriminative ability that falls just below the conventional 0.70 benchmark for acceptable discrimination. The calibration curve (Figure 4b) demonstrated a high degree of agreement between the predicted probabilities and the observed event rates. The curve closely tracked the ideal diagonal line, suggesting that the model is well-calibrated and its risk estimates are reliable.

### 3.5 Feature importance and SHAP analysis

**Figure 5:**
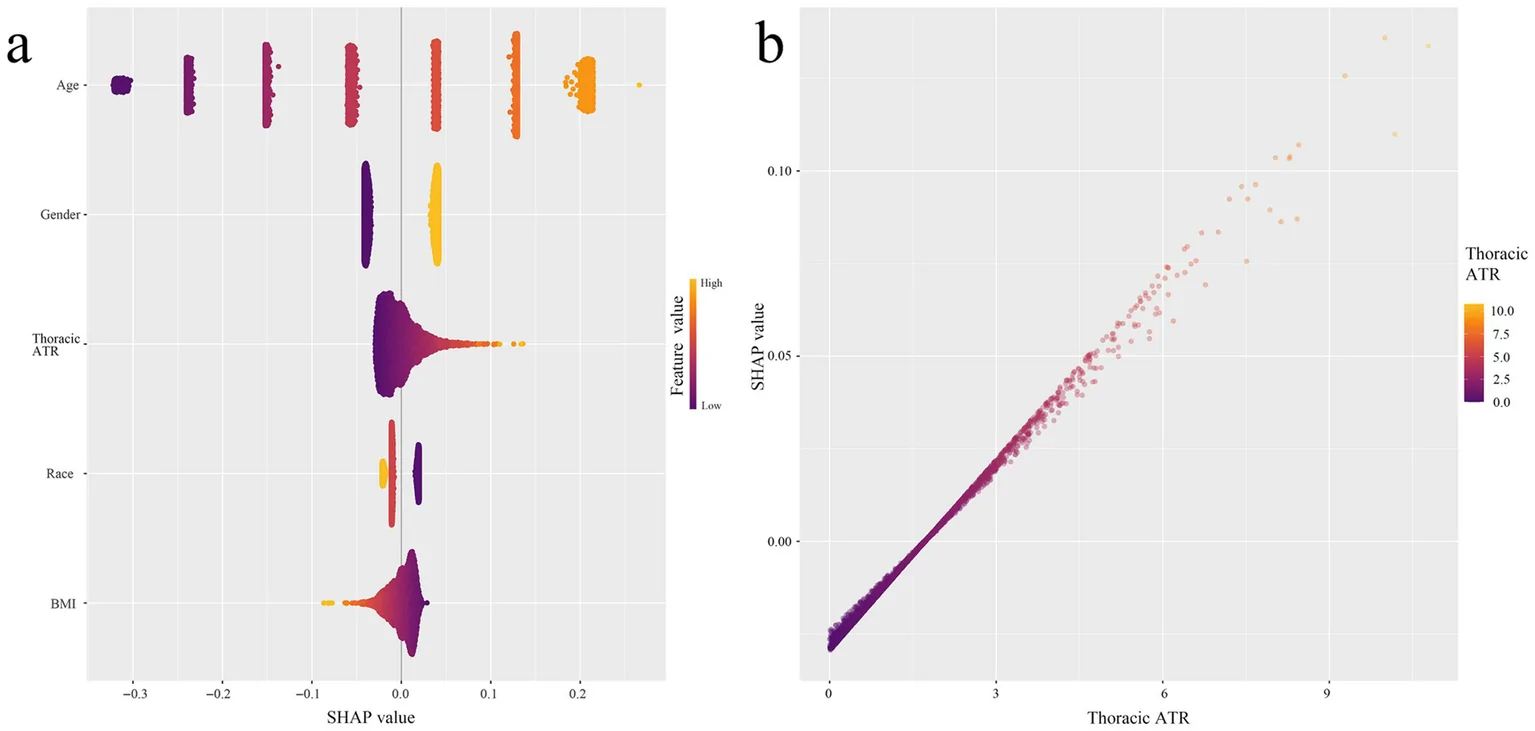
SHapley Additive exPlanations (SHAP) model interpretation. **(a)** SHAP summary plot displaying the global feature importance. Each dot represents a participant, with the color indicating the feature value (purple for low, yellow for high). Features are ranked on the *y*-axis based on their mean absolute SHAP values, showing that Age, Gender, and Thoracic angle of trunk rotation (ATR) are top contributors to the model. **(b)** SHAP dependence plot for Thoracic ATR. The *x*-axis represents the Thoracic ATR value, and the *y*-axis represents the corresponding SHAp value (i.e., the contribution of ATR to the log-odds of myopia for each individual). The near-linear appearance of this plot is an expected mathematical property of applying SHAP to a logistic regression model, in which the feature contribution is proportional to the feature value after centering. Importantly, the direction and slope of this relationship confirm a consistent positive association: Higher Thoracic ATR values correspond to greater predicted probability of myopia across the full range of observed values, further validating ATR as a meaningful concurrent morphological marker.

We utilized SHAP analysis to visually interpret the contribution of each feature to the model’s predictions. As shown in the SHAP summary plot (Figure 5a), Thoracic ATR emerged as a critical feature associated with myopia, ranking third in overall feature importance, trailing only age and gender. This ranking highlights that spinal posture (ATR) adds significant informative value beyond basic demographic factors. The SHAP dependence plot (Figure 5b) revealed a clear monotonic positive correlation. As the Thoracic ATR value increases (*x*-axis), the SHAP value (contribution to the log-odds of myopia) rises (*y*-axis), confirming that elevated trunk rotation is consistently associated with an increased probability of myopia.

## 4 Discussion

The overall prevalence of myopia in this study (52.8%) reflects the severe myopia burden among school-aged children in rural China (24, 30). This study aimed to explore the independent concurrent association between thoracic ATR (a sensitive indicator of early subclinical spinal asymmetry) and myopia among school-aged children from a rural county in northeastern China. It addresses a gap in prior research, which predominantly focused on severe, clinically diagnosed scoliosis rather than early subclinical postural variations. The key results demonstrate that thoracic ATR is independently associated with myopic status: 1° increase in ATR was associated with an approximately 7% higher odds of myopia after adjusting for confounders, and children in the highest tertile of ATR had a 23% elevated odds compared to the lowest tertile, with a linear association observed.

Preliminary comparisons with existing literature show consistency in the broader link between musculoskeletal health and myopia (15, 16). However, key differences distinguish this research. Discrepancies with some prior studies (13) may stem from their reliance on diagnosed scoliosis (Cobb angle ≥10°, capturing later-stage deformity), whereas this study’s ATR measurement sensitively detects early, subclinical trunk asymmetry. Rather than framing postural deviation as a strict predictor of myopia, this study views subclinical ATR as a concurrent epidemiological marker capturing the shared consequences of poor physical ergonomics. Furthermore, although this association is statistically significant, the effect size (OR = 1.07) is relatively modest, and the model achieved only modest discrimination (AUC = 0.685); ATR is therefore best interpreted as one of several concurrent markers rather than as a standalone screening indicator. This implies that subclinical spinal asymmetry is merely one component of a complex, multifactorial system and cannot serve as a standalone screening determinant for visual health.

In this context, the present findings add to recent Asian school-based evidence by showing that the association between myopia and spinal asymmetry may be detectable even below the conventional threshold for clinically suspected scoliosis. While previous studies have mainly examined clinically diagnosed scoliosis or scoliosis-screening positivity, our analysis focused on subclinical thoracic ATR and identified a graded positive association with myopia. This pattern is broadly consistent with the Shanghai cohort and the joint spinal-refractive examination study, supporting the possibility that early trunk asymmetry may be part of a wider vision–posture risk profile in school-aged children.

The mechanism underlying the association between thoracic ATR and myopia is not yet fully understood, and three pathways have been proposed in the literature. Among these, the shared-behavioral-ergonomics hypothesis is, in our judgment, the most plausible explanation for the modest effect size observed in this cross-sectional design. Prolonged near-work in habitually asymmetric postures simultaneously imposes asymmetric mechanical loading on the spine and asymmetric accommodative-vergence demand on the visual system (9, 10); this dual-exposure account is parsimonious, consistent with our finding that ATR contributes informatively beyond age and gender, and aligns with recent evidence from a 9,583-student Shanghai middle-school cohort showing that scoliosis screening positivity and poor reading/writing posture are independently associated with myopia (15). Importantly, this shared-exposure interpretation does not exclude a reverse or reciprocal pathway: children with myopia may shorten their working distance or adopt compensatory head tilt and asymmetric sitting during near tasks, which could secondarily contribute to postural imbalance. Thus, the observed association should be interpreted as potentially bidirectional or mutually reinforcing, rather than as evidence that thoracic ATR necessarily precedes myopia. The genetic-pleiotropy hypothesis, based on rare deleterious variants such as SLC16A8 that link retinal glucose metabolism with skeletal development (14), is more directly relevant to severe early-onset high myopia accompanied by structural scoliosis than to the subclinical ATR range observed here. The autonomic-nervous-system pathway, linking spinal mechanoreceptors to autonomic regulation of accommodation and axial elongation (31–33), remains the most speculative, as it relies on a mechanistic chain (spinal mechanoreceptor to autonomic tone to ciliary/choroidal regulation) that has not been demonstrated directly in pediatric populations. We therefore consider shared ergonomic exposure the leading working hypothesis, while acknowledging that prospective longitudinal and mechanistic studies will be required to disentangle these pathways.

Subgroup analysis showed that the association between thoracic ATR and myopia appeared more pronounced in female and Han students, although all interaction tests were non-significant. These subgroup patterns should therefore be regarded as exploratory and do not establish true effect modification. In particular, the apparently stronger association among female students may partly reflect baseline sex differences in myopia prevalence or chance variation, rather than a genuine sex-specific ATR effect. In a national retinal-clinic-based study from Bhutan, Rai et al. reported that among patients presenting to the country’s national vitreo-retinal services, the prevalence of myopia was highest among urban females, followed by urban males, rural females, and rural males, suggesting that sex and living environment may both contribute to myopia-related patterns (34). In the present study, myopia was also more common among female students; however, because the sex interaction was not statistically significant, the female subgroup finding should be interpreted cautiously. Pubertal maturation may also influence ATR and postural asymmetry, but direct pubertal indicators were not collected; this explanation therefore remains speculative and warrants evaluation in future longitudinal studies.

Prior work has consistently shown that ATR varies systematically with age, sex, and skeletal growth phase during childhood and adolescence (35, 36), and that statistically significant differences between asymmetry-free and asymmetric subgroups emerge most clearly between 9 and 11 years of age—a window that overlaps with early pubertal onset, particularly in girls (37). Postural asymmetry indicators such as ATR should therefore be interpreted in the context of biological maturation rather than chronological age alone.

Furthermore, our machine learning approach (SHAP analysis) provided a global interpretation of feature importance, ranking Thoracic ATR as the third most critical feature associated with myopia, following age and gender. This alignment between the machine learning interpretation and the traditional regression results reinforces the conclusion that thoracic spinal asymmetry is a non-negligible concurrent marker for myopia, independent of other demographic confounders.

From a public-health translational perspective, the practical value of detecting subclinical thoracic ATR alongside refractive screening is twofold. First, ATR measurement is rapid, low-cost, non-invasive, and requires only brief examiner training, which makes it readily implementable in resource-limited rural school health programs. Second, detecting subclinical ATR may identify children who would otherwise pass conventional scoliosis screening but who nonetheless carry a possible dual-system risk profile involving both visual and musculoskeletal health. On this basis, we propose a tiered management framework: (i) Children with ATR ≥ 5° should be referred for orthopaedic evaluation according to established scoliosis-screening practice; (ii) Children in the highest ATR tertile (cut-point >1.86) may be prioritized for closer school-based follow-up and periodic re-screening, with concurrent refractive monitoring. This cut-point is sample-specific and should not be interpreted as a universal clinical threshold; and (iii) Children below this range should receive non-medical ergonomic education, including parental and teacher guidance on desk-and-chair fit, posture breaks during reading and writing, and integration with existing outdoor-time recommendations for myopia prevention. We emphasize that the tertile cutoff used here is sample-specific and should be re-derived in each implementation population rather than treated as a universal threshold.

This study has several limitations. First, the cross-sectional design cannot establish whether thoracic ATR precedes or causes myopia; for the same reason, we cannot determine whether the magnitude of ATR or its increase over time is more closely associated with myopia onset or progression. Prospective longitudinal studies with repeated ATR and refractive measurements are therefore needed. Second, myopia was assessed using non-cycloplegic autorefraction, which may overestimate myopia prevalence due to residual accommodation, particularly in younger children. To reduce this potential misclassification, a more conservative threshold of SE ≤ −0.75 D was adopted instead of the conventional −0.50 D cutoff. Nevertheless, future studies using cycloplegic refraction are needed to confirm the prevalence estimates and validate the observed association. Third, thoracic ATR values in the present study sample were predominantly within the subclinical range and below commonly used scoliometer referral thresholds. Therefore, the findings should not be interpreted as evidence regarding clinically suspected scoliosis or structural spinal deformity. Rather, they apply mainly to subclinical trunk rotational asymmetry, which was the intended focus of this epidemiological study. Fourth, important behavioral factors related to myopia, including outdoor time, screen time, near-work duration, reading distance, and lighting conditions, were not collected. Therefore, residual confounding or mediation by shared ergonomic and lifestyle exposures cannot be excluded. Future longitudinal studies incorporating these behavioral measures are needed. Fifth, direct indicators of pubertal development were not collected, and chronological age can only serve as an indirect proxy for biological maturation. In an age-proxy sensitivity analysis, the association remained positive across all subsets but lost statistical significance in the two youngest, smallest subsets, where the reduced sample size limited precision; these chronological proxies cannot definitively classify individual pubertal status. Therefore, residual confounding or effect modification by pubertal maturation cannot be fully excluded, and future longitudinal studies should incorporate direct maturation indicators. Sixth, because data were drawn from a single rural county in northeastern China, the findings should be interpreted as region-specific evidence. Future multi-site studies sampling rural populations across different geographic regions of China will be required to evaluate national representativeness. Seventh, formal inter- and intra-examiner reliability were not directly evaluated within the school-aged sample; residual pediatric-specific measurement variability cannot be excluded.

## 5 Conclusion

This study identifies a significant association between subclinical thoracic ATR elevation and increased odds of myopia among school-aged children in a rural county of northeastern China. Rather than implying strict causality, the findings suggest that minor thoracic rotational asymmetry serves as an important concurrent marker of myopic status, reflecting a dual-health vulnerability likely driven by shared environmental factors such as prolonged near-work in poor posture. From a public health perspective, these findings advocate for integrating simple, non-invasive spinal posture assessments, such as ATR measurement, into routine pediatric vision screenings, enabling early ergonomic interventions to safeguard both ocular and musculoskeletal health in school-aged children. The conclusions should be regarded as region-specific evidence rather than as nationally representative inference, and multi-site studies sampling rural populations across different regions of China are required to assess wider generalizability.

## Data Availability

The datasets presented in this article are not readily available because they contain health data from minors, and the ethics approval and guardian consent obtained for this study do not permit public sharing of individual-level data. Requests to access the datasets should be directed to Weiwei Fu, fuww@sibet.ac.cn.

## References

[1] Holden BA Fricke TR Wilson DA Jong M Naidoo KS Sankaridurg P . Global prevalence of myopia and high myopia and temporal trends from 2000 through 2050. Ophthalmology. (2016) 123:1036–42. doi: 10.1016/j.ophtha.2016.01.006, 26875007

[2] Maruyama T Yotsukura E Torii H Mori K Inokuchi M Tokumura M . Children in Tokyo have a long sustained axial length from age 3 years: the Tokyo myopia study. J Clin Med. (2022) 11:4413. doi: 10.3390/jcm11154413, 35956029 PMC9369597

[3] Karuppiah V Wong L Tay V Ge X Kang L. School-based programme to address childhood myopia in Singapore. Singapore Med J. (2021) 62:63–8. doi: 10.11622/smedj.2019144, 31680176 PMC8027142

[4] Lee Y Lee J An D Seo KY Yoon S Congdon N. Rising burden of myopia among south Korean young adults based on 13-year trends, associated factors, and projections to 2050. Sci Rep. (2026) 16:2371. doi: 10.1038/s41598-025-32048-0, 41398351 PMC12816136

[5] Lakawicz JM Bottega WJ Fine HF Prenner JL. On the mechanics of myopia and its influence on retinal detachment. Biomech Model Mechanobiol. (2020) 19:603–20. doi: 10.1007/s10237-019-01234-1, 31650370

[6] Tan NYQ Sng CCA Jonas JB Wong TY Jansonius NM Ang M. Glaucoma in myopia: diagnostic dilemmas. Br J Ophthalmol. (2019) 103:1347–55. doi: 10.1136/bjophthalmol-2018-313530, 31040131

[7] Jonas JB Ang M Cho P Guggenheim JA He MG Jong M . IMI prevention of myopia and its progression. Invest Ophthalmol Vis Sci. (2021) 62:6. doi: 10.1167/iovs.62.5.6, 33909032 PMC8083117

[8] Wolffsohn JS Kollbaum PS Berntsen DA Atchison DA Benavente A Bradley A . IMI – clinical myopia control trials and instrumentation report. Invest Ophthalmol Vis Sci. (2019) 60:M132–60. doi: 10.1167/iovs.18-25955, 30817830

[9] Lin Z Gao TY Vasudevan B Ciuffreda KJ Liang YB Jhanji V . Near work, outdoor activity, and myopia in children in rural China: the Handan offspring myopia study. BMC Ophthalmol. (2017) 17:203. doi: 10.1186/s12886-017-0598-9, 29149871 PMC5693484

[10] Li C Zhao Y Yu Z Han X Lin X Wen L. Sagittal imbalance of the spine is associated with poor sitting posture among primary and secondary school students in China: a cross-sectional study. BMC Musculoskelet Disord. (2022) 23:98. doi: 10.1186/s12891-022-05021-5, 35090408 PMC8800310

[11] Cai Z Wu R Zheng S Qiu Z Wu K. Morphology and epidemiological study of idiopathic scoliosis among primary school students in Chaozhou, China. Environ Health Prev Med. (2021) 26:71. doi: 10.1186/s12199-021-00989-3, 34217201 PMC8254979

[12] Gallego-Siles JR Siles-Fuentes MJ Ibáñez-Vera AJ Cortés-Pérez I Obrero-Gaitán E Lomas-Vega R. Idiopathic scoliosis in subjects with eye diseases: a systematic review with meta-analysis. Ann N Y Acad Sci. (2024) 1533:81–8. doi: 10.1111/nyas.15102, 38327125

[13] Cai J Zhou Y Chen X Huang X Li L Zhu Y . Is refractive error a factor affecting scoliosis? PLoS One. (2024) 19:e0303324. doi: 10.1371/journal.pone.0303324, 38739623 PMC11090344

[14] Wen W Zhao Z Zheng Z Zhao S Zhao H Cheng X . Rare variant association analyses reveal the significant contribution of carbohydrate metabolic disturbance in severe adolescent idiopathic scoliosis. J Med Genet. (2024) 61:666–76. doi: 10.1136/jmg-2023-109667, 38724173 PMC11228217

[15] Wang Y Yang D Zhang F Qi W Lu Q Wu H . Analysis of the association between reading and writing postures and comorbidity of myopia and scoliosis in junior middle school students. Front Pediatr. (2025) 13:1576575. doi: 10.3389/fped.2025.1576575, 41040640 PMC12488097

[16] Luo J Xie L Luo Y Cai Z Wang X Wang C . The association between spinal health and visual function in a pediatric population: insights from large-scale health examinations. Front Public Health. (2026) 13:1702548. doi: 10.3389/fpubh.2025.1702548, 41648767 PMC12868225

[17] Bunnell WP. An objective criterion for scoliosis screening. J Bone Joint Surg Am. (1984) 66:1381–7. doi: 10.2106/00004623-198466090-00010, 6501335

[18] Prowse A Pope R Gerdhem P Abbott A. Reliability and validity of inexpensive and easily administered anthropometric clinical evaluation methods of postural asymmetry measurement in adolescent idiopathic scoliosis: a systematic review. Eur Spine J. (2016) 25:450–66. doi: 10.1007/s00586-015-3961-7, 25917824

[19] Bunnell WP. Outcome of spinal screening. Spine. (1993) 18:1572–80. doi: 10.1097/00007632-199309000-00001, 8235833

[20] von Elm E Altman DG Egger M Pocock SJ Gøtzsche PC Vandenbroucke JP . The strengthening the reporting of observational studies in epidemiology (STROBE) statement: guidelines for reporting observational studies. Ann Intern Med. (2007) 147:573–7. doi: 10.7326/0003-4819-147-8-200710160-00010, 17938396

[21] Pan CW Ramamurthy D Saw SM. Worldwide prevalence and risk factors for myopia: prevalence and risk factors for myopia. Ophthalmic Physiol Opt. (2012) 32:3–16. doi: 10.1111/j.1475-1313.2011.00884.x 22150586

[22] Flitcroft DI He M Jonas JB Jong M Naidoo K Ohno-Matsui K . IMI – defining and classifying myopia: a proposed set of standards for clinical and epidemiologic studies. Invest Ophthalmol Vis Sci. (2019) 60:M20. doi: 10.1167/iovs.18-25957, 30817826 PMC6735818

[23] Senoo Y Furuse T Hasebe S. The optimal cut-off value of non-cycloplegic autorefraction for diagnosing myopia in school-aged children. Jpn J Ophthalmol. (2022) 66:455–60. doi: 10.1007/s10384-022-00928-x, 35788446

[24] Zhang J Li Z Ren J Wang W Dai J Li C . Prevalence of myopia: a large-scale population-based study among children and adolescents in Weifang, China. Front Public Health. (2022) 10:924566. doi: 10.3389/fpubh.2022.924566, 35958863 PMC9358211

[25] Marshall WA Tanner JM. Variations in pattern of pubertal changes in girls. Arch Dis Child. (1969) 44:291–303. doi: 10.1136/adc.44.235.291, 5785179 PMC2020314

[26] Marshall WA Tanner JM. Variations in the pattern of pubertal changes in boys. Arch Dis Child. (1970) 45:13–23. doi: 10.1136/adc.45.239.13, 5440182 PMC2020414

[27] Sun Y Tao F Su PY China Puberty Research Collaboration. National estimates of pubertal milestones among urban and rural Chinese boys. Ann Hum Biol. (2012) 39:461–7. doi: 10.3109/03014460.2012.712156, 22862548

[28] Shu W Zong X Li H. Secular trends in age at pubertal onset assessed by breast development among Chinese girls: a systematic review. Front Endocrinol. (2022) 13:1042122. doi: 10.3389/fendo.2022.1042122, 36506059 PMC9729541

[29] Hu J Han W Zhou M Geng Y Zhang J Zhou F . Secular trends in the median age at menarche and spermarche among chinese children from 2000 to 2019 and analysis of physical examination indicators factor. Am J Hum Biol. (2025) 37:e24198. doi: 10.1002/ajhb.24198, 39653583

[30] Dong L Kang YK Li Y Wei WB Jonas JB. Prevalence and time trends of myopia in children and adolescents in China: a systemic review and meta-analysis. Retina. (2020) 40:399–411. doi: 10.1097/IAE.0000000000002590, 31259808

[31] Oakley PA Moustafa IM Harrison DE. The influence of sagittal plane spine alignment on neurophysiology and sensorimotor control measures: optimization of function through structural correction. In: Bernardo-Filho M da Cunha de Sá-Caputo D Seixas A Taiar R, editors. Therapy Approaches in Neurological Disorders. London: IntechOpen; (2021). Chapter 4.

[32] Ashwini DL Raju TR. Autonomic nervous system and control of visual function. Ann Neurosci. (2023) 30:151–3. doi: 10.1177/09727531231176119 37779550 PMC10540761

[33] Li M Xu Q Yan X Wang J Xiang Y. Relationship between autonomic nervous system activity and axial length in children. Med Sci Monit. (2023) 29:e939451. doi: 10.12659/MSM.939451, 37177779 PMC10190173

[34] Rai BB Ashby RS French AN Maddess T. Rural-urban differences in myopia prevalence among myopes presenting to Bhutanese retinal clinical services: a 3-year national study. Albrecht Von Graefes Arch Klin Exp Ophthalmol. (2021) 259:613–21. doi: 10.1007/s00417-020-04891-6 32803328

[35] Grivas TB Vasiliadis ES Koufopoulos G Segos D Triantafyllopoulos G Mouzakis V. Study of trunk asymmetry in normal children and adolescents. Scoliosis. (2006) 1:19–26. doi: 10.1186/1748-7161-1-19, 17137516 PMC1693569

[36] Hajka A Walatek J Myśliwiec A Lipowicz A. Scoliotic posture and biological age measured by pubertal growth spurt in adolescent boys and girls. Anthropol Anz. (2023) 80:247–54. doi: 10.1127/anthranz/2023/1634, 37165669

[37] Adamczewska K Wiernicka M Malchrowicz-Mośko E Małecka J Lewandowski J. The angle of trunk rotation in school children: a study from an idiopathic scoliosis screening. Prevalence and optimal age screening value. Int J Environ Res Public Health. (2019) 16:3426. doi: 10.3390/ijerph16183426, 31527403 PMC6765789

