# Supplementary material for: Thoracic angle of trunk rotation and its association with myopia among schoolchildren in a rural county of northeastern China
Source: Front Public Health. 2026 Aug 6;14:1836894. doi: 10.3389/fpubh.2026.1836894 (PMC13490146; doi:10.3389/fpubh.2026.1836894)
Supplement: Supplementary file 1 [file Table_1.DOCX]

Supplementary Material

# 1 Supplementary Table

**Supplementary Table 1.** Variance inflation factors before and after refinement of Model 3

| **Variable** | **Original Model 3**  *Gender + Age + Ethnicity + Waist + Hip + BMI* | **Revised Model 3**  *Gender + Age + Ethnicity + BMI* |
| --- | --- | --- |
| Female (sex) | 1.08 | 1.00 |
| Age | 1.51 | 1.02 |
| Ethnicity  (highest VIF among Manchu / Others dummy variables) | 1.23 | 1.23 |
| Waist circumference | 5.12 | — |
| Hip circumference | 6.00 | — |
| BMI | 1.03 | 1.02 |
| Thoracic ATR | 1.01 | 1.01 |

*Note: Variance inflation factors (VIFs) were calculated using the vif() function in the R package car, applied to the linear predictor of each logistic regression model. A VIF > 5 is conventionally regarded as indicating substantial multicollinearity, and a VIF > 10 as severe multicollinearity. In the original Model 3, waist circumference (VIF = 5.12) and hip circumference (VIF = 6.00) exceeded the conventional threshold, indicating notable collinearity among anthropometric covariates. After retaining BMI as the sole anthropometric covariate, all VIFs in the revised Model 3 were below 2, confirming that multicollinearity was no longer a concern.*

*Abbreviations: BMI, body mass index; ATR, angle of trunk rotation; VIF, variance inflation factor.*

**Supplementary Table 2.** Univariate analysis of myopia

| **Variable** | **OR (95% CI)** | ***p*** |
| --- | --- | --- |
| Gender, n (%) |  |  |
| Male | Reference |  |
| Female | 1.40 (1.20~1.63) | <0.001 |
| Age (year) | 1.46 (1.39~1.54) | <0.001 |
| Ethnicity, n (%) |  |  |
| Han | Reference |  |
| Manchu | 0.83 (0.70~0.99) | 0.038 |
| Others ^a^ | 0.83 (0.65~1.05) | 0.127 |
| Waist circumference (cm) | 1.02 (1.01~1.03) | <0.001 |
| Hip circumference (cm) | 1.03 (1.02~1.04) | <0.001 |
| BMI | 1.02 (1.00~1.04) | 0.016 |
| Thoracic ATR | 1.11 (1.05~1.18) | <0.001 |
| Thoracic ATR group |  |  |
| Less than 0.86 | Reference |  |
| 0.86 to 1.86 | 1.28 (1.05~1.53) | 0.013 |
| More than 1.86 | 1.37 (1.14~1.66) | 0.001 |

*Note: OR (95% CI) values represent effect-size estimates from univariate logistic regression models; P values are derived from Wald tests.*

*Abbreviations: BMI, body mass index; ATR: angle of trunk rotation, measured by the Bunnell scoliometer, OR: odds ratio; CI, Confidence interval.*

*^a^ includes Xibe, Mongolian, Chinese Korean, Tujia, Daur, Yi, Tibetan, Zhuang nationality.*

**Supplementary Table 3.** Sensitivity analysis of thoracic ATR rotation direction (fully adjusted for gender, age, ethnicity, and BMI)

| **Analysis** | **Result** |
| --- | --- |
| Direction alone (positive vs. negative rotation) | OR = 0.96, 95% CI 0.82–1.14, P = 0.661 |
| \|ATR\| × Direction interaction in fully adjusted model | Interaction OR = 0.98, 95% CI 0.87–1.11, P = 0.805 |
| ATR effect within positive-rotation subgroup (n = 1,518) | OR = 1.06 per 1°, 95% CI 0.97–1.16, P = 0.186 |
| ATR effect within negative-rotation subgroup (n = 1,078) | OR = 1.08 per 1°, 95% CI 0.99–1.18, P = 0.075 |

*Note: This sensitivity analysis used the original signed thoracic ATR records (n = 2,596 after excluding three participants with exactly zero thoracic rotation). At the screening stage, signed thoracic ATR values were recorded to encode the direction of rotation (i.e., the laterality of the rib prominence on forward bending, corresponding to clockwise vs. counterclockwise rotation). The primary analysis used the absolute magnitude (|ATR|); this sensitivity analysis used the original signed values. Rotation direction alone was not associated with myopia, the |ATR| × direction interaction was not significant, and the per-degree |ATR| effect was directionally consistent across the two rotation-direction subgroups. These findings support the methodological appropriateness of using |ATR| in the primary model and indicate that the observed ATR–myopia association reflects the magnitude of rotational asymmetry rather than its laterality.*

*Abbreviations: ATR, angle of trunk rotation; |ATR|, absolute magnitude of thoracic ATR; OR, odds ratio; CI, confidence interval; BMI, body mass index.*

**Supplementary Table 4.** Sensitivity analyses using sex-specific chronological-age proxies for pubertal maturation

| **Sample** | **N** | **Events, n**  **(%)** | **Age-proxy threshold** | **Model 3 OR**  **(95% CI) per 1° thoracic ATR** | **P value** |
| --- | --- | --- | --- | --- | --- |
| Original full sample (main analysis) | 2,599 | 1,373 (52.8%) | — | 1.07 (1.01–1.14) | 0.023 |
| Lenient age-proxy subset | 2,463 | 1,275 (51.8%) | girls <12 y, boys <13 y | 1.09 (1.02–1.16) | 0.012 |
| Moderate age-proxy subset | 2,005 | 957 (47.7%) | girls <11 y, boys <12 y | 1.09 (1.02–1.17) | 0.018 |
| Stricter age-proxy subset | 1,505 | 623 (41.4%) | girls <10 y, boys <11 y | 1.07 (0.99–1.17) | 0.083 |
| Exploratory very-young age-proxy subset | 618 | 183 (29.6%) | girls <8 y, boys <9 y | 1.04 (0.91–1.19) | 0.565 |

*Note: These thresholds were used as sex-specific chronological-age proxies for pubertal maturation and should not be interpreted as definitive Tanner-stage classification. Model 3 adjusted for gender, age, ethnicity, and BMI. OR (95% CI) values are effect-size estimates from logistic regression; P-values are derived from Wald tests. The estimate in the very-young subset should be interpreted cautiously because of the substantially reduced sample size and number of myopia events.*

*Abbreviations: ATR, angle of trunk rotation; OR, odds ratio; CI, confidence interval; BMI, body mass index.*
